# A brief review of mRNA therapeutics and delivery for bone tissue engineering

**DOI:** 10.1039/d2ra00713d

**Published:** 2022-03-22

**Authors:** Arun Kumar Rajendran, Sivashanmugam Amirthalingam, Nathaniel S. Hwang

**Affiliations:** School of Chemical and Biological Engineering, The Institute of Chemical Processes, Seoul National University Seoul 08826 Republic of Korea nshwang@snu.ac.kr; Interdisciplinary Program in Bioengineering, Seoul National University Seoul 08826 Republic of Korea; Bio-MAX/N-Bio Institute, Institute of Bio-Engineering, Seoul National University Seoul 08826 Republic of Korea; Institute for Engineering Research, Seoul National University Seoul 08826 Republic of Korea

## Abstract

The therapeutics for bone tissue regeneration requires constant advancements owing to the steady increase in the number of patients suffering from bone-related disorders, and also to find efficient and cost-effective treatment modalities. One of the major advancements in the field of therapeutics is the development of mRNAs. mRNAs, which have been extensively tested for the vaccines, could be very well utilized as a potential inducer for bone regeneration. The ability of mRNAs to enter the cells and instruct the cellular machinery to produce the required native proteins such as BMP or VEGF is a great way to avoid the issues faced with growth factor deliveries such as the production cost, loss of biological function *etc.* However, there have been a few hurdles for using mRNAs as an effective therapeutic agent, such as proper dosing, tolerating the degradation by RNases, improving the half-life, controlling the spatio-temporal release and reducing the off-target effects. This brief review discusses the various developments in the field of mRNA therapeutics especially for bone tissue engineering, how nano-formulations are being developed to effectively deliver the mRNAs into the cells by evading the immune responses, how researchers have developed certain strategies to increase the half-life, to successfully deliver the mRNAs to specific bone defect area and bring about effective bone regeneration.

## Abstract 2

This review provides an update on the various mRNA preparations, carriers that can deliver these preparations and how they could potentially enhance the bone regeneration.

## Graphical abstract

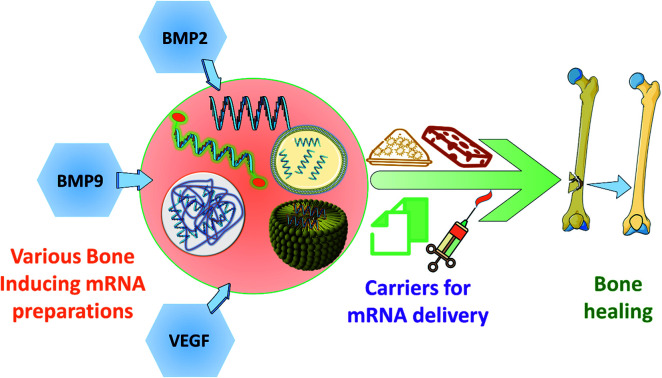

## 1 Introduction

Bone is a highly complex and dynamic tissue with numerous functions of protecting the various vital organs, providing mobility, a site for the production of blood cells, providing structural stability and acting as a storage reservoir of various minerals.^1,2^ Bone is endowed with good regenerative capacity in comparison with other organs in the human body. However, large and complex bone defects, bone-related metabolic disorders and various genetic anomalies require effective and supportive treatment strategies for aiding regeneration. Currently, various tissue engineering and regenerative strategies with the use of 3-dimensional scaffolds are being utilized to circumvent the limitations of autograft and allografts.^2^ At present, various means of preparing scaffolds are proposed with the use of natural and synthetic polymers for improving the bone regeneration. Additionally, to enhance bone regeneration, cells, growth factors and bioactive molecules including hormones (parathyroid, steroids, estrogens), statins and anti-resorptive drugs (*e.g.*, bisphosphonates and strontium ranelate) are included in the scaffolds.^3^ Generally, osteoinductive growth factors, such as bone morphogenetic protein 2 (BMP2), are considered to be potent molecules for enhancing bone regeneration. In 2002, the first commercial device containing BMP2 was marketed for clinical use.^4^ However, supra-physiological loading of BMP2 (to avoid premature degradation, rapid release, and systemic circulation) lead to many off-target side effects including osteolysis, inflammation, retrograde ejaculation and so on.^4,5^ Moreover, the large scale synthesis and logistics of clinical-grade growth factors is an expensive affair. To avoid the above-mentioned problems, researchers turned towards gene delivery to cells and the first clinical trial on transplanting transfected cells started as early as the 1990s.^6^ However, many concerns exist in gene therapy including the use of viral vectors for transfection, overexpression of target gene, transfection in non-dividing cells, random genome insertion and carcinogenicity.^7^ Recently, as a safe alternative to gene therapy, mRNA therapy has gained substantial interest among researchers. mRNA therapy has many advantages over DNA, for example, mRNA exerts its function in the cytosol, eliminating the nuclear entry, thereby avoiding the risk of random genome insertion. mRNA delivery can be carried out using non-viral vectors (such as lipids and polymers) and provides very good transfection efficiency, whereas viral vectors are required for high transfection efficiency in DNA therapeutics.^8,9^

There have been constant advancements in utilizing mRNA as a therapeutic material and it has been discussed elaborately by various authors.^8–11^ The principles such as mRNA designing, mRNA packing for improvising the delivery has gone through various iterations of research leading to well understood concepts.^10,12^ Researchers also work on various strategies to increase the transfection efficiency, improvise on the immune stealth and efficient delivery to specific site, as discussed in the literature.^11–18^ This review briefly discusses and provides an update on how the mRNA therapeutics have evolved over time, the various strategies that are being explored to overcome the bottlenecks faced in utilizing mRNA as efficient therapeutic aids, the integration of bone tissue engineering biomaterials with mRNA for better localized delivery and some of the novel methods including the co-delivery of mRNA, for producing mRNA protecting proteins, thereby, reducing the need for chemical modifications in mRNA. Apart from mRNAs for bone tissue regeneration, the review also gives an overview about the future possibilities of utilizing the mRNA therapeutics for treating various bone related genetic disorders.

## 2 mRNA delivery principles

In 1969, mRNA was successfully transcribed in a cell-free system for the first time.^12^ From then, four major applications of mRNA delivery can be considered: protein replacement, immunotherapy for cancer and other infectious diseases, gene editing and regenerative applications.^10^ Delivery of the mRNA can be achieved either by physical methods such as microinjection,^13^ gene gun^14^ and electro-transfection^15^ or by chemical methods including cationic polymers,^16^ lipoplexes^17^ and lipid nanoparticles.^18^ Even though electroporation provides effective means of delivering mRNA into the cytosol, it is of high cost and an inconvenient procedure for *in vivo* conditions, preventing the clinical translation.^11^ In comparison with physical methods, chemical methods are widely used due to the convenience in administration to patients, cost-effectiveness and multi-functionality. Of all the chemical methods, encapsulating mRNA in lipid nanoparticles is the most effective method till date, as it overcomes many technical barriers for effectively delivering mRNA. Of note, COVID-19 mRNA vaccine candidates such as mRNA-1273 and BNT162b2 were based on lipid nanoparticles.^19–21^

For the successful delivery of mRNA, three key aspects need to be considered. They are the protection of mRNA, mRNA dosing and optimizing the release kinetics.

### 2.1 mRNA protection

**Fig. 1:**
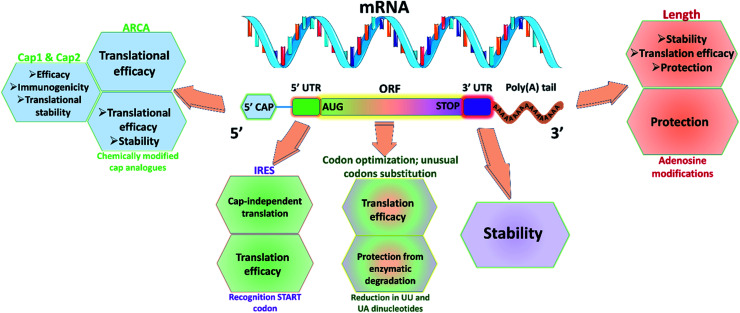
Representative scheme of cmRNA containing five structural elements: 5′ capping structure with anti-reverse cap analog (ARCA), 5′-untranslated region (UTR) with internal ribosomal entry sites (IRES), open reading frame (ORF) with AUG in kozak sequence as start codon, 3′-UTR and poly(A) tail for improved translation efficiency, stability, half-life and reduced immunogenicity. Usage of modified nucleotides (such as 5-methylcytidine (5mC), 5-methyluridine (5mU), *N*6-methyl adenosine (5mA), pseudouridine (Ψ), *N*1-methylpseudouridine (N1mΨ), and 2-thiouridine (s2U)) would help in evading immune activation.

Utmost care needs to be taken for protecting mRNA from premature degradation, denaturation and maintaining the bioactivity inside cells. It could be majorly achieved through developing chemically modified mRNA (cmRNA) and employing certain complexation agents. The cmRNA is synthesized through *in vitro* transcription (IVT), therefore it is sometimes referred to as synthetic IVT mRNA. The generic structure of cmRNA contains a 5′-cap, 5′-untranslated region (UTR), open reading frame (ORF), 3′-URF and poly(A) tail (Fig. 1). However, the rules of modification are open-ended and many researchers are working on them to improve the stability and translation efficiency.

#### 2.1.1 5′-Cap

Recent understanding of native mRNA has taught many aspects for designing cmRNA. Eukaryotic native mRNA possesses a cap structure at 5′ end. The cap0 is formed by the conjunction of inverted 7-methyl guanosine (7mG) and the first nucleotide.^22^ The cap in the cmRNA can affect the stability and translation in two ways. First, the cap in the cmRNA has to bind with eukaryotic translation initiation factor 4E (eIF4E) to initiate translation.^23^ Second, it prevents degradation from various exonucleases present in the cell. Recently, new cap structures were found in native mRNA namely, cap1 and cap2, which were formed by the methylation of the second and third nucleotide at 2′O or 3′O position.^24^ These structures are found to be less immunogenic and increase translation efficiency, compared to cap0. Thus, to bio-mimic the native mRNA many strategies were developed to incorporate these cap structures in cmRNA. Recently, TriLink BioTechnologies has developed a method to incorporate cap1 or cap2 in the mRNA at a claimed efficiency of 94% using enzymatic capping technique.^22^ Additionally, Elangovan *et al.* synthesized cmRNA of BMP-2 with anti-reverse cap analog (ARCA) 7-methyl (3′-*O*-methyl) guanosine to prevent the elongation of cmRNA in a wrong direction during IVT process.^10,16^ Another aspect to be considered is the presence of decapping enzymes in the cytosol, which could decap the cmRNA leading to degradation that causes an immunogenic reaction. To circumvent such problems, researchers have utilized chemically modified cap analogs in cmRNA to improve the half-life. These modifications include O-to-NH imidodiphosphate, O-to-BH_3_ boranophosphate, O-to-S phosphorothioate and non-bridging oxygen.^25^

#### 2.1.2 UTRs

Even though UTRs don't participate in the translation, their length and sequence impart a vital role in regulating translation and protein expression. Specifically, 5′-UTR helps in the initiation of mRNA translation, whereas, 3′-UTR is involved in stabilizing mRNA and the extent of translation.^26^ To improve the cmRNA translation, it is imperative to incorporate internal ribosomal entry sites (IRES) as they could initiate the translation even when capping sites are absent. By this, when eIF4E expression is low, the cmRNA will be translated as it contains IRES.^27^ To improve the stability and extent of protein translation, many studies have utilized specific sequences from α and β-globin genes. The length of the 3′ UTRs may dictate the protein's subcellular localization. Longer 3′-UTRs could lead to expression of proteins in the cell membrane, whereas shorter 3′-UTRs could promote translation in the endoplasmic reticulum.^28^

#### 2.1.3 Poly(A) tail

Native eukaryotic mRNA has poly(A) tail at their 3′ end for stability and translation. It is essential to add optimal length of poly(A) tail in the cmRNA for the above-mentioned reasons. It could be added either co-transcriptionally or by post-transcription using enzymatic addition. The optimal length of poly(A) tail depends on the target site, as the optimal length is approximately 100 bases for human epithelial cells, whereas around 120 bases are required for dendritic cells for improving the translational efficiency, stability and half-life.^11,29,30^

#### 2.1.4 ORF

In addition to the 5′-cap and 3′ poly(A) tails, the ORF sequence design could directly impact the efficiency of translation and immunogenicity of mRNA. A decrease in the number of UU and UA dinucleotides within the ORF was found to protect the cmRNA from the decapping enzymes. The start codon (5′-AUG-3′) in cmRNA should be part of a Kozak sequence 
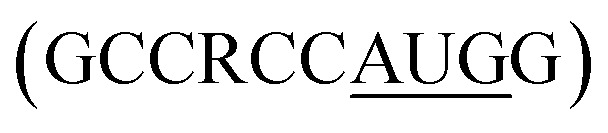
 for higher translation efficiency. Other start codons showed lower translation efficiency compared to AUG.^22,31^ Researchers have discovered that cmRNA sequences can dictate the formation of secondary structures, which can influence the degradation of the cmRNA by hydrolysis. Recently, computationally sophisticated algorithms that design optimal cmRNA structures to reduce overall hydrolysis rate have been developed for improved cmRNA stability.^32^

#### 2.1.5 Modified nucleosides

Exogenous mRNA could be detected by toll-like receptors (TLR) and retinoic acid-inducible protein 1 (RIG1) interpreting it as a sign of viral infection, activating the immune system, and thus leading to degradation of cmRNA. To circumvent this problem, pseudo nucleotides can be incorporated in cmRNA to reduce the immunogenicity markedly by evading TLRs and activation of RIG1.^33^ Some of the modified nucleotides that avoid the activation of TLRs include: 5-methylcytidine (5mC), 5-methyluridine (5mU), *N*6-methyl adenosine (5mA), pseudouridine (Ψ), *N*1-methylpseudouridine (N1mΨ), and 2-thiouridine (s2U). Additionally, Ψ and s2U can evade homing from RIG1.^21,34^ Additionally, these modified nucleotides in cmRNA can protect from RNAse activity, thereby increasing the half-life of cmRNA.^35^ Elangovan *et al.* synthesized cmRNA of BMP-2 with Ψ and 5mC modification in its codon sequence for reducing the immunogenicity. 100% substitution of Ψ and 5mC for uridine and cytosine didn't elicit interferon-α (IFN-α) secretion in comparison to the cmRNA with unmodified nucleotides, thereby showing reduced immunogenic reaction for the modified nucleotides.^16^ Incorporation of these modified codons could affect the incorporation efficiency of RNA polymerase during the IVT process and also reduce the decoding speed and accuracy. Balmayor *et al.* synthesized cmRNA of BMP-2 with 25% incorporation of 5mC and s2U for cytosine and uridine.^36^ Thus, by carefully modifying the mRNAs, we could develop strategies to overcome the practical shortcomings and develop an efficient mRNA therapy for bone regeneration.

### 2.2 mRNA dosing

Although the chemical modification and alteration of cmRNA can increase translation efficiency, the biological half-life of cmRNA is short compared to the healing time required for bone injuries, which can last for weeks rather than days. To circumvent this shortcoming, carefully designed bone-substituting biomaterials can be used for sustained delivery of cmRNA nanocomplexes. cmRNA dosing is very critical, as it should be sufficiently low to cause immune reaction and at the same time, should provide a therapeutic effect. However, very few studies have focused on cmRNA dosing and many studies need to be carried out for clinical translation. Recently, Vega *et al.* studied the bone healing in critical-sized defect in rat femur bone using cmRNA of BMP-2 loaded in collagen sponges.^37^ The authors observed a dose-dependent increase in bone healing. Specifically, 50 μg of cmRNA of BMP-2 displayed complete bone healing where torque-to-failure was similar to normal femur bone, while lower doses, 5 μg and 10 μg of cmRNA could stimulate *de novo* bone formation but not sufficient to bridge the critical-sized defects. At 25 μg, there was only half the samples showed complete union. Thus, a clear dose dependency was observed in bone healing. Geng *et al.* utilized cmRNA of BMP-2 and VEGF for transfecting BMSCs.^38^ Here, the authors utilized 1 : 1 ratio of cmRNA of BMP-2 and VEGF for driving osteogenic and angiogenic expression, thereby resulting in synergetic bone formation. Although, researchers are coming up with new strategies for designing cmRNA, additionally, studies need to be carried out for studying the half-life of cmRNA when implanted in the defect site. Further, dose regime needs to be studied in osteoporotic, glucocorticosteroids induced avascular bone necrosis of bone, genetic and metabolic disorders.

### 2.3 Spatio-temporal release kinetics

Bone regeneration is a highly complex process that involves various growth factors acting spatio-temporally. It is important to mimic natural bone healing in order to achieve successful bone regeneration in large and complex bone defects.^3,39^ So far, researchers have not determined whether the timing or duration of cmRNA-encoded BMP-2 impacts bone regeneration. Nevertheless, it is important to understand the timing and release kinetics of cmRNA encoded for various proteins in bone regeneration. Taking cues from the previous studies of growth factors delivery in bone regeneration, it has been suggested that a spatio-temporal release favors enhancement in bone regeneration.^40–43^ To achieve a spatio-temporal control, 3-D biomaterial scaffold-based delivery systems would be a key factor, as a 3-D matrix will also provide support for regeneration in large bone defects. The degradation kinetics and mechanical properties of the scaffold are beyond the topic of interest to be covered in this review and the readers may look at reviews focused on those topics for further understanding.^44–47^ Another reason for controlling the undesirable spatial release of BMP-2 is to prevent the numerous side-effects of BMP-2 that have been reported.^4,5^ Acri *et al.* focused on alleviating the off-target effects of cmRNA of BMP-9 by capping with salicylic acid poly(anhydride-ester) (SAPAE).^48^ Salicylic acid released from the degradation of the polymer could inhibit bone formation. Further studies on SAPAE could potentially help in reducing the off-target effects of cmRNA in the future.

## 3 mRNA delivery strategies

**Fig. 2:**
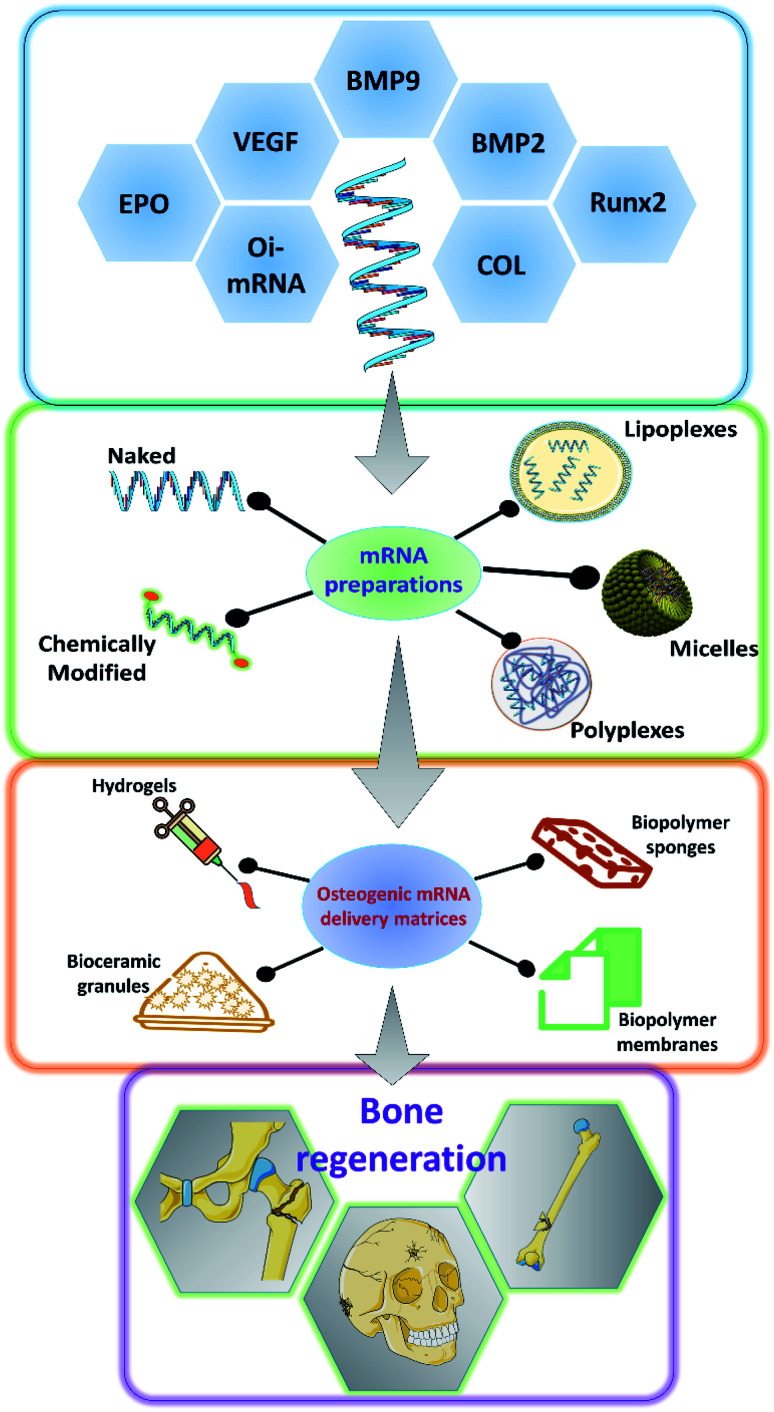
Schematic showing the various osteogenic proteins for which mRNAs could be potentially encoded, the ways in which, the synthesized mRNA could be formulated for the application, the various carrier matrices that are being explored to carry the mRNA formulations for effective bone regeneration. BMP: bone morphogenetic protein; COL: collagen; Runx2: runt related transcription factor 2; VEGF: vascular endothelial growth factor; EPO: erythropoietin; Oi-mRNA: osteoinductive mRNAs.

As it has been discussed earlier and by various authors, mRNA has a great potential to be used in a variety of applications including bone tissue engineering.^49,50^ There has been a steady advancement in the design and manufacturing process of mRNA as a therapeutic agent in the field of medicine. However, owing to the innate nature of the mRNA, such as its relatively larger size than microRNAs or oligonucleotides, the rapid degradation of mRNA even before it reaches the cellular compartments and the preservation of translational efficiency proves to be a great challenge in the stable delivery of mRNA therapeutics.^51–54^ Researchers have explored various ways to overcome these hurdles to effectively deliver the mRNA for therapeutic applications (Fig. 2). Some of such strategies that have been developed are discussed below.

### 3.1 Encapsulation and complexation

One of the well-proven ways to improve the efficiency of the delivery of mRNA particles is by encapsulating them using various chemicals including but not restricted to polymers, lipids and dendrimers.^53^ The utilization of cationically charged lipids and lipid-like materials as an encapsulating and delivery medium for mRNA has been one of the oldest and well-studied.^55–57^ Lipids were utilized as early as 1978 by Dimitriadis *et al.*, to encapsulate mRNA in the form of liposomes.^58^ Further with the development of Lipofectin®, which is an equimolar combination of 1,2-dioleoyl-*sn*-glycero-3-phosphoethanolamine (DOPE) and *N*-[1-(2,3-dioleoyloxy)propyl]-*N*,*N*,*N*-trimethylammonium chloride (DOTMA), transfection of nucleic acids was rapidly improved.^59–62^ Improving upon this, various lipids and lipid derivatives such as cholesterol, DLinDMA, cKK-E12, TT3 and MPA-A have been used in conjunction with DOPE to prepare encapsulations for delivering mRNAs.^63–69^ Lipids and lipid-based nanoparticles can be made into various forms of encapsulation devices such as liposomes, lipid nanoparticles (LNPs), microbubbles, micelles, lipid implants and emulsions.^35,49,70–72^ Lipoplexes has been utilized to deliver hBMP2-cmRNA for enhancing bone regeneration.^73^ Similarly, it has also been shown that bone tissue can be induced by delivering BMP2/NS1 mRNAs by loading into lipopolyplexes.^74^ Apart from lipid and lipid-derived nanoparticles, researchers have alternatively explored various cationic polymers to pack the mRNAs. Such delivery vehicles can be synthesized from various polymer categories such as polyamines, block polymers and polypeptides. Polyethylenimine (PEI) is one of the commonly used polymers which can effectively condense the nucleic acid, thus providing a suitable delivery vehicle.^75,76^ PEIs can aid in endosomal escape, as they could act as a proton (H^+^) sponge, thus absorbing the acid moieties when inside the endosomal cavity.^77,78^ This would lead to osmotic imbalance and rupture of the endosome thus releasing the polyplex with the mRNA unharmed. However, this effect has been under debate by various researchers.^79^ Leng *et al.*, designed and synthesized an osteoinductive-mRNA-PEI complex and showed that this could effectively enhance bone regeneration.^76^ Similarly, other researchers have utilized biopolymers such as chitosan, alginate along with other transfecting agents to deliver the mRNAs.^80–84^ However, not much work has been carried out specifically for controlling bone regeneration.

### 3.2 Hydrogel based delivery systems

Hydrogels are networks of polymers that could absorb and entrap large quantities of water. Hydrogels are so versatile that they could be made into various forms according to the specific needs. They have been extensively utilized in the field of tissue engineering, especially for bone tissue regenerations and has served as a great carrier for various small molecules, growth factors, proteins, drugs, nucleic acids, genes and cells targeted towards enhancing bone regeneration.^85–89^ The water absorbed between the polymer networks serve as potentially favorable reservoirs for the above mentioned therapeutic agents. Researchers also have a wide array of polymers to choose from depending on their needs and the type of therapeutic agent that is going to be delivered. For example, hydrogels based on fibrin, calcium phosphates, PLGA, collagen and similar biodegradable materials could serve as excellent matrices for bone regeneration.^90^ Furthermore, the hydrogels are usually delivered to the specific site, thus providing an advantage of local delivery of the intended mRNA particles. Thus, utilizing the bone regeneration aiding hydrogel matrices to carry mRNAs that could promote the osteodifferentiation would be a two-sided advantage and should enhance the bone regeneration compared to regular hydrogels. The hydrogels could also be tuned for the specific spatio-temporal release of the mRNA particles. This strategy was used to load BMP2-cmRNA on micro & macro biphasic calcium phosphate granules and these granules were then delivered using fibrin-based hydrogel scaffold.^91^ It was shown that this could efficiently induce the osteogenic pathways in the rat bone marrow-derived stem cells. Similarly, lipoplexes carrying chemically modified RNA towards hBMP2 were loaded into fibrin hydrogels.^36^ These hBMP2-cmRNA loaded hydrogels were able to induce osteogenesis in stem cells and also exhibited rapid healing of femoral defects in rats. Similarly, hydrogels comprising of alginate and/or chitosan have been utilized to deliver lipoplexes encapsulating Cy3-hGLuc-mRNAs.^82^ Such systems are potentially utilized to carry various mRNAs that would be of great therapeutic effect towards bone tissue regeneration. Furthermore, there have been various hydrogel formulations designed and synthesized from different biocompatible polymers such as collagen, hyaluronic acid, Matrigel and PEG, which were used to deliver miRNAs and siRNAs encapsulated in lipofectamine, silver nanoparticles and PEI to facilitate bone regeneration.^81,92–95^ Although these systems majorly used miRNAs and siRNAs, these could be easily replaced with mRNAs for achieving similar results for bone regeneration.

### 3.3 mRNA delivery *via* scaffolds

Scaffolds serve as temporary structures for some of the key functions and milestones during tissue regeneration such as defect filling, attachment sites for cells, temporary surfaces for the cellular establishment and artificial matrices which could be subsequently replaced by native extracellular matrix, and also to serve as reservoirs for nutrients. Scaffolds are usually made from biocompatible and biodegradable materials such as polymers, ceramics, polysaccharides, proteins and a combination of these, based upon the necessity of the target tissue that is being regenerated.^96–98^ There have been numerous fabrication techniques that have been well established to synthesize scaffolds ranging from simple cast-drying to 3-D printing techniques.^98–100^ Scaffolds could also be fabricated in such a way that various small molecules, drugs, growth factors, DNA, RNA and peptides could be embedded into them through either physical entrapment, covalent linking and non-covalent linkages.^101,102^ These versatile properties make scaffolds a very good carrier for mRNA delivery. By choosing an appropriate composition of biodegradable polymers, we can tune the degradation rate of the scaffolds, which in turn could help us in controlling the dose and temporal release of the loaded mRNA particles. Collagen scaffolds have been often used for bone tissue regeneration as collagen forms the organic component of the bone and researchers have utilized such scaffolds to deliver the mRNAs for BMP2 production. PEI has been used to encapsulate BMP2-cmRNA or BMP9-cmRNA and further embedded into the collagen scaffolds for enhancing bone regeneration. It has been found that such a system could enhance the regeneration of defects in femoral bone in rats. Lipoplexes carrying the BMP2-cmRNA have also been dispersed in collagen scaffolds to stimulate bone growth in critical-sized mid-femoral defects in rodents. A dual mRNA delivery of BMP2 and VEGF-A was applied using lipoplexes in collagen sponge for enhancing bone regeneration as well as angiogenesis in rat cranial defects.^38^ This dual delivery strategy could overcome one of the most commonly faced limitations of scaffolds failure due to the lack of vasculature. Similarly, collagen membrane, collagen sponges have been utilized to carry mRNA lipoplexes or polyplexes of BMP2 or BMP9 for enhancing bone tissue regeneration.^103,104^ Researchers were also able to prolong the shelf life of the hBMP2 encoding mRNAs by loading it onto collagen sponges and vacuum-drying it.^105^ They have shown that these mRNAs loaded scaffolds were stable up to 6 months at room temperature and also stimulate protein production for up to 6 days. Recently researchers have utilized a dual delivery of mRNAs in which mRNA coding for protein NS1 can help in immune evasion and thus can protect the BMP2 mRNA from the immune system from degradation. Thus, when mRNA lipo-polyplexes for both BMP2 & NS1 were loaded onto collagen-nanohydroxyapatite scaffolds, they could enhance the translation of osteogenic BMP2, leading to better *in vivo* bone formation.^106^ Thus by utilizing scaffolds in an advantageous way to prolong the shelf life, spatio-temporally control the mRNA release, to carry multiple target encoding mRNAs we could be able to utilize the mRNAs as a great therapeutic aid for bone tissue engineering in the future.

## 4 mRNA delivery in bone regeneration

The first study on delivery cmRNA for tissue regeneration application was studied in 2015, incidentally, it was on bone regenerative application and thereafter many studies followed up.^16,49^ There are two approaches to delivering cmRNA for regeneration, namely the first approach is to transfect the mesenchymal stem cells. The transfected cells were loaded in the 3-D scaffold matrix and implanted in the defect region for regeneration. The second approach is to directly load the cmRNA encapsulated/complexed particles into the scaffold and implant them for regeneration. The latter approach is convenient and many studies utilized this approach for the therapeutic application. Elangovan *et al.* synthesized cmRNA of BMP-2 and complexed it with PEI to get polyplex.^16^ The polyplexes were incorporated into the collagen sponge and the bone regeneration potential in the rat calvarial defect model was evaluated. The results showed that the cmRNA loaded scaffold showed higher bone formation compared to plasmid DNA encoded for BMP-2. In their follow-up study, researchers studied the bone regeneration potential of cmRNA of BMP-2 and BMP-9.^107^ Results displayed a 2-fold increase in bone connectivity density for the BMP-9 group compared to BMP-2. Similarly, in another follow-up study, Khorsand *et al.* loaded the PEI-cmRNA of BMP-9 complex in collagen membrane for bone regeneration.^108^ Balmayor *et al.* developed lipoplexes with cmRNA of BMP-2, loaded in fibrin scaffold and studied the bone regeneration in rat femur bone defect model for 2 weeks.^36^ The results suggested an improved bone formation compared to the fibrin matrix. Plasmid linearization was carried out using *Not*I and *Xba*I and the transfection efficiency studies displayed a higher efficiency for *Not*1 cleaved cmRNA, as the length of 3′UTR gets varied, which in turn affected the stability and transfection efficiency. The authors claim that with the use of *Not*I cleaved cmRNA, the quantity of cmRNA required for *in vivo* formation could be reduced, as the authors used 2.5 μg of cmRNA compared to the 25 μg of cmRNA used in Elangovan *et al.* study.^16,36^ However, direct comparison in the animal model is warranted to ascertain the claim. In a follow-up study from the group, researchers improved the stability of the cmRNA of BMP-2 by changing the substitution of modified nucleotides.^104^ It includes, 35% uridine being replaced with 5-iodo-uridine and 7.5% of cytidine had been replaced with 5-iodo-cytidine. Further, a short translator initiator sequence and poly(A) sequence after the AU rich region were added at 5′ UTR. These modifications improved the stability and transfection efficiency with a reduced immunogenic response. Badieyan *et al.* studied the storage stability of collagen sponge loaded with lipoplex of cmRNA of BMP-2.^17^ The prepared scaffold was ready to use in the clinical scenario and showed storage stability at room temperature up to 6 months without reduction in cmRNA activity. The authors discussed the unsuitability of PEI polyplex for freeze-drying technique and therefore, *in situ* addition of PEI polyplex is the only way out.

Ceramic particles are commercially used for bone regeneration. Combining the ceramic particles in the scaffold would improve the mechanical property and help in osteogenic differentiation.^109,110^ Balmayor *et al.* developed transcript-activated matrices (TAM) made of fibrin, micro-macro biphasic calcium phosphate and cmRNA of BMP-2.^91^ The study showed that TAM was able to increase the osteocalcin and collagen gene expression with high mineral deposition. Utzinger *et al.* developed injectable calcium phosphate cement (CPC) containing PLGA microparticles encapsulating cmRNA of BMP-2/lipoplex for bone regeneration.^111^ PLGA microparticles degraded rapidly to allow the release of cmRNA and provide the space for cell infiltration. It is a promising approach to combine the cmRNA with ceramic cement, as they would provide early regeneration compared to conventional CPC. In addition, transcript technology can be utilized for improving the osseointegration of titanium (Ti) implants. Fayed *et al.* studied the cmRNA coating strategies on Ti using three different biomaterials (poly-d,l-lactic acid, fibrinogen and fibrin).^112^ All the biomaterial coated Ti samples showed improved transfection efficiency with noticeable improvement in fibrinogen and fibrin coated samples.

**Fig. 3:**
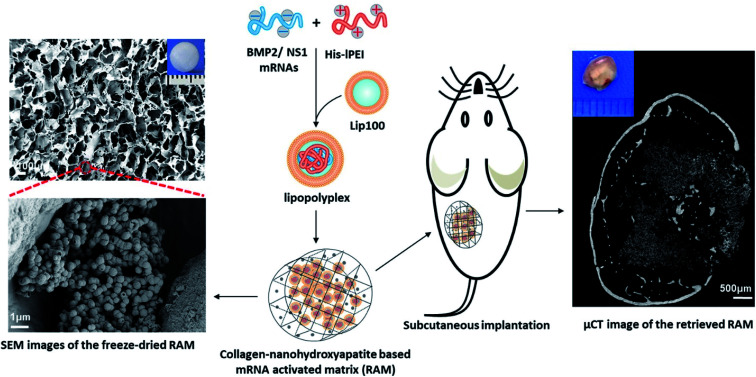
Schematic showing an advanced delivery of dual mRNAs, using lipopolyplexes loaded into the collagen-nanohydroxyapatite matrix, one for mRNA protective protein and another for BMP2 production resulting in enhanced bone formation. This figure has been reproduced from ref. 106 with permission from Elsevier, copyright 2021.

The high cost of HPLC purification in modified nucleotide and other associated issues like reduced decoding speed and fidelity can be circumvented by co-delivering non-structured protein-1 (NS1) mRNA, which would evade the immune response by forming a complex with RIG1, thereby blocking the activation of nuclear factor kappa B (NFκB) and interferon regulatory protein, resulting in the reduction of interferons.^113^ It was also found that co-delivering NS1 increases the transfection efficiency by suppressing the interferon synthesis. Thus, a new approach of using mRNA for NS1 takes away the requirement of using modified nucleotides for evading the immune response. Recently, Wang *et al.*, co-delivered mRNA of NS1 and BMP-2 using Lipofectamine in C3H10T1/2 cell line for improving osteogenic differentiation.^114^ In this study, researchers showed that naive nucleotides could be used in mRNA for therapeutic purposes without eliciting an immune reaction by delivering mRNA of BMP-2 with NS1. Additionally, *in vitro* studies displayed that the co-delivering strategy enhanced the osteogenic markers and calcium deposition. In a follow-up study, researchers assessed the bone regeneration potential of this strategy in subcutaneous implantation of lipopolyplexes loaded in the collagen-nanohydroxyapatite scaffold in mice.^106^ The results showed a *de novo* bone formation after weeks of subcutaneous implantation (Fig. 3). For the clinical translation, orthotropic bone formation studies and dose regimen for co-delivering of NS1/BMP-2 mRNA in large animals would pave the way.

Geng *et al.* utilized the cell-based system, wherein the cells are transfected with cmRNA and the transfected cells were implanted in the bone defects, as so to reduce cytotoxic effects of cationic lipids/polymers during direct delivery.^38^ Here, the authors delivered cmRNA of BMP-2 and VEGF for simultaneous enhancement of osteogenesis and angiogenesis, thereby achieving higher bone regeneration. Uchida *et al.* prepared cmRNA of RUNX2 and transfected mesenchymal spheroids for osteogenic differentiation.^115^ Transfected cells could be used in the future for transplantation.

## 5 Clinical perspectives

mRNA therapeutics have come a long way and has been well established for various clinical treatments. Since mRNAs, if properly delivered, can bring about very effective protein transcription at a cellular level, it serves as a very effective tool in treating various diseases, including some of the genetic related disorders. This is mainly because, when mRNAs are delivered successfully into a cell, they can effectively command the cellular machinery to produce a fully functional protein, which can bring about various intended biological functions.^116^ This is much more cost-effective and efficient than delivering the protein itself for the treatment. For example, genetic mutation of disorders such as alkaptonuria (ochronosis), in which patient's body is deficient in homogentisic acid oxidase, could lead to calcified intra-articular loose bodies, which severely compromises the quality of life of the patients.^117,118^ In such a scenario, mRNA can be designed to synthesize the homogentisic acid oxidase enzyme and upon successful delivery, it will enable the cells to produce the same, thus offering a therapeutic outcome. A similar strategy could be utilized to treat patients suffering from osteopetrosis, in which osteoblasts are defective in producing macrophage colony-stimulating factors due to mutations in various genes, which leads to improper osteoclast differentiation, thus leading to improper remodeling of bone.^119,120^ Herein, mRNAs can be targeted towards osteoblasts and instructions can be coded, program the osteoblasts to synthesize macrophage colony-stimulating factors, thus leading to proper osteoclast formation and normal bone remodeling. Similarly, mRNAs can be directed for the production of osteoprotegerin, a protein that can act as the controller of bone resorption. The mutation in the TNFRSF11B gene can cause deficiency of osteoprotegerin leading to hereditary hyperphosphatasia.^121,122^ mRNAs that could help the production of osteoprotegerin would be a great strategy to treat such a hereditary disorder.

Apart from the utilization of mRNAs in metabolic or genetic bone disorders, a similar approach could be effectively used to improve bone healing, as discussed in the examples in previous sections. mRNAs could be utilized to control several signaling pathways, such as BMP signaling, production of RUNX2, BMP and so on, that are essential for osteogenic differentiation.^123^ This approach of using the mRNAs to enhance bone regeneration would be great in patients wherein, their innate bone regeneration abilities are reduced due to age-related factors, underlying medical conditions and various other factors. When supplemented with the currently utilized scaffolds and ceramics for bone regeneration, mRNAs could play a substantial role in expediting the bone regeneration process, thus improving the quality of life of the patients. This effect has been proven in various *in vivo* animal models, wherein mRNAs were delivered along with the fibrin, collagen or other similar biomaterials. With the approvals and effective utilization of mRNA vaccines for COVID-19, human society has much more awareness about the efficacy of mRNA for prophylactic purposes now, which was lacking before.^124^ The dilemmas and the fears that were once lurking for using mRNAs for therapeutics in humans is slowly reducing and thus it would be a great start to push forward the mRNAs as a viable and effective treatment, especially for bone tissue regeneration in the upcoming years.

## 6 Challenges

The various advantages of using mRNAs for bone tissue regeneration has been discussed. Although the future looks promising for mRNAs to be potentially utilized as therapeutic aids, several challenges still had to be overcome to become a majorly preferred treatment modality. mRNAs have been successfully deployed and proven as effective vaccines for human use. However, their utility in the treatment of genetic or metabolomic diseases needs much more research. Although it is now established that off-target effects can be avoided when using mRNAs, the current administration of mRNA is mostly systemic, therefore will result in diffusion in most of the tissues. For example, an mRNA injection targeted to improve the production of BMP for enhancing bone defect healing would also act on normal bone tissues, thus resulting in unwanted bone turnover in an otherwise normal bone. A similar systemic effect could be expected when mRNAs are being delivered to induce the production of various other proteins which might be needed locally but might have deleterious effects on rather normal tissues. This could be minimized by designing mRNAs that work only in the specific diseased cells, and also through local delivery systems such as hydrogels or scaffolds. Furthermore, the dose of the mRNA delivered also has to be carefully adjusted depending on the population.^125^ Another issue faced with the usage of mRNA therapeutics is that the delivered mRNAs have a very short half-life. This is attributed to the presence of RNases which are present throughout the body.^52,54^ Therefore, care has to be taken that, mRNA formulations are not degraded faster when injected into the body. Although researchers are utilizing various techniques such as encapsulation, chemical modifications and/or addition of adjuvants, there is a long way to standardize an easy-to-go solution for effective delivery of mRNA therapeutic agents for all scenarios. Apart from the above-mentioned issues, evading immunogenicity is another challenging aspect. Usually the mRNA those are being delivered could be easily mistaken for viral infection by the body's innate immune cells and could also cause inflammation. Researchers try to circumvent this issue in different ways such as decreasing the number of U content in the mRNA sequences, nucleotide modifications, addition of poly(A) tails, and purification of mRNA to remove the dsRNAs.^53^ Furthermore, care has to be taken during these processes such that it does not alter the potency or the intended protein expression capabilities of the delivered mRNA. With the advancements going on, and the standardization of manufacturing protocols, we could hope that researchers could overcome these challenges in the near future, to provide a better and more effective mRNA therapeutic solution for the masses.^126^ With the involvement of numerous pharma companies, we could also expect that these processes could fasten up and will be available to the public very soon.^127^

## 7 Conclusion

mRNAs have been emerging and evolving as a really powerful tool in the world of therapeutics. The potential applications where they could be utilized are getting diversified day by day, ranging from the vaccine, protein replacements, cancer immunotherapy, genome editing and also in regenerative strategies. With researchers designing newer delivery platforms that could evade the host's immune responses, increasing the longevity of the particles in the system, designing mRNA sequences with better protein translational efficiencies, purifying the mRNA, improving target specificity, enhancing the pharmacological effect and controlling the immunogenicity, delivering the mRNAs locally using scaffolds/hydrogels, improving the biodegradability and clearance, we could expect the field to grow much larger in a positive way. Furthermore, with the more number of clinical trials that are underway for exploring the efficacy of mRNA in various therapeutic applications, including regenerative therapies, and with the improved awareness among the society regarding the efficacy of mRNA therapies, it could be said with confidence that mRNA based bone regeneration will be practiced often in the foreseeable future.

## Conflicts of interest

There are no conflicts to declare.

## Supplementary Material
